# Nationwide Case-Control Analysis of Merkel Cell Carcinoma and Associated Skin Cancer Comorbidities: An Examination of the All of Us Database

**DOI:** 10.7759/cureus.54426

**Published:** 2024-02-18

**Authors:** Kritin K Verma, Travis S Dowdle, Sumoni Verma, Tejas P Joshi, Richard F Wagner

**Affiliations:** 1 Dermatology, Texas Tech University Health Sciences Center, Lubbock, USA; 2 Dermatology, University of Texas Medical Branch, Galveston, USA; 3 Science, St. Andrew's Episcopal School, Austin, USA; 4 Dermatology, Baylor College of Medicine, Houston, USA

**Keywords:** national institutes of health, all of us database, case-control analysis, skin cancer comorbidities, merkel cell carcinoma

## Abstract

Introduction

Merkel cell carcinoma is an aggressive neuroendocrine tumor that is related to immunosuppression and the Merkel cell polyomavirus. It is more common on the head and neck and has been associated with other skin malignancies such as basal cell carcinoma, squamous cell carcinoma, and melanoma. However, there has never been a nationwide investigation that quantifies Merkel cell carcinoma's connection with these subgroups.

Methods

Utilizing the National Institutes of Health's All of Us open-access database, a retrospective study was conducted by filtering for Merkel cell carcinoma through the International Classification of Diseases, 9th and 10th Clinical Modification codes 209.* and C4A.*, respectively. This led to the inclusion of 41 patients in the study, with each instance compared to four control patients without merkel cell carcinoma, matched by age, race, and gender. The data's demographics and skin cancer co-morbidities were collected and evaluated with odds ratios and 95% confidence intervals using Wald's method.

Results

In patients with merkel cell carcinoma, a statistically significant gradient of increasing risk for developing basal cell carcinoma (Odds Ratio, 11.63; 95% Confidence Interval, 4.30-31.45; P < 0.0001), squamous cell carcinoma (Odds Ratio, 15.09; 95% Confidence Interval, 3.87-58.84; P = 0.0001), and melanoma (Odds Ratio, 27.94; 95% Confidence Interval, 3.26-239.48; P = 0.0024) was observed. The race/ethnicity demographics showed that 85.4% of the patients were white, and they were at the highest risk of developing merkel cell carcinoma. However, the study has limitations, such as the inability to identify the stage of merkel cell carcinoma among patients and the lack of consideration for other confounding variables.

Conclusion

The study examines the link between merkel cell carcinoma and other skin malignancies, underscoring the need for more national research to better understand the underlying causes that contribute to this link. The findings also indicate the possibility of sample bias in the All of Us database, emphasizing the need to assess the patient population's representativeness in such investigations.

## Introduction

Merkel Cell Carcinoma (MCC) is an aggressive neuroendocrine tumor that usually occurs in elderly Caucasian males, is associated with immunosuppression, and is linked to an underlying infection by the Merkel cell polyomavirus (MCPyV). It primarily manifests on the head and neck [1]. MCC has also been associated with basal cell carcinoma (BCC), squamous cell carcinoma (SCC), and melanoma [1,2]. However, a nationwide study has not been previously conducted. Therefore, utilizing the National Institutes of Health's All of Us open-access database [3], we aimed to quantify MCC’s association with the above-listed subgroups.

## Materials and methods

Filtering for MCC through the International Classification of Diseases (ICD), 9th and 10th Clinical Modification (CM) codes 209.* and C4A.*, respectively, through the electronic medical record searching function on the All of Us database in October 2023 led to the inclusion of 41 patients in our study. No exclusion was done beyond the inclusion criteria in regards to MCC cases obtained. Patients' age was reported as the age of diagnosis. Each instance of MCC was compared to four control patients matched by age, race, and gender, with exclusions made for control patients diagnosed with ICD-9-CM and ICD-10-CM codes 209.* and C4A.*, respectively. The data's demographics and skin cancer comorbidities were collected and evaluated with odds ratios (ORs) and 95% confidence intervals (CIs) using Wald's method.

## Results

The diagnosed age of MCC in Table 1 determined that the majority of patients with MCC are above the age of 65, representing 65.85% of the MCC patient population. Additionally, most cases of MCC are notably male (Table 1).

**Table 1:** Comparison of MCC Diagnosed Age and Number of Patients Enrolled Based on ICD-10-CM Code C4A.* and ICD-9-CM Code 209.*. MCC: Merkel Cell Carcinoma

Age Range for MCC Diagnosed (years)	Cases (%) (n = 41)	Male Sex at Birth (%)
<50	2 (4.88)	2 (100.00)
≥50 to <65	12 (29.27)	9 (69.23)
≥65	27 (65.85)	24 (92.31)

In patients with MCC, there is a statistically significant gradient of increasing risk for developing BCC (OR, 11.63; 95% CI, 4.30-31.45; P < 0.0001), SCC (OR, 15.09; 95% CI, 3.87-58.84; P = 0.0001), and melanoma (OR, 27.94; 95% CI, 3.26-239.48; P = 0.0024) (Table 2).

**Table 2:** Case-Control Analysis of Demographics and Skin Cancer Comorbidities in Patients with MCC in the All of Us Database. *Statistically significant values between cases and control, defined at <0.05. CI, Confidence Interval; OR, odds ratio; SD, Standard Deviation; MCC, Merkel Cell Carcinoma

Demographics and Comorbidities of patients with Merkel Cell Carcinoma and controls in the All of Us Database					
Chraractaristic		Cases (%) (n = 41)	Controls (%) (n = 164)	OR (95% CI)	P value
Average age, SD (years)		68.1, 9.67	68.1, 9.67	-	1.00
Male		33 (78.8)	132 (78.8)	-	1.00
Race/Ethnicity	White	35 (85.4)	140 (85.4)	-	1.00
	Black	4 (9.76)	16 (9.76)	-	1.00
	Hispanic	0 (0.00)	0 (0.00)	-	1.00
	Asian	0 (0.00)	0 (0.00)	-	1.00
	Other	2 (4.88)	8 (4.88)	-	1.00
Basal Cell Carcinoma		14 (34.15)	7 (4.27)	11.63 (4.30-31.45)	< 0.0001*
Squamous Cell Carcinoma		9 (22.0)	3 (1.83)	15.09 (3.87-58.84)	0.0001*
Melanoma		6 (14.6)	1 (0.609)	27.94 (3.26-239.48)	0.0024*
Dermatofibrosarcoma Protuberans		0 (0)	0 (0)	-	-
Atypical Fibroxanthoma		0 (0)	0 (0)	-	-
Undifferentiated Pleomorphic Sarcoma		0 (0)	0 (0)	-	-
Sebaceous Carcinomas		0 (0)	0 (0)	-	-
Extra Mammary Paget's Disease		0 (0)	0 (0)	-	-
Microcystic Adnexal Carcinoma		0 (0)	0 (0)	-	-

In Table 2, we attempted to analyze additional tumors, with the total number of patients in the database displayed after each, such as dermatofibrosarcoma protuberans (4), atypical fibroxanthoma (2), undifferentiated pleomorphic sarcoma (24), sebaceous carcinomas (3), extramammary Paget's disease (8), and microcystic adnexal carcinoma (2), regrettably, insufficient case/control data was obtained. The scarcity of data may derive from the recent database establishment and indicates an area necessitating further research.

## Discussion

The underlying association between MCC and other types of skin cancer may be explained primarily by the degree of UV exposure, age, and Fitzpatrick skin type [4], but again, the degree of association on a national scale has not been previously explored. The race/ethnicity demographics concur with these findings, as 35 (85.4%) white patients were at the highest risk of developing MCC (Table 2).

Our retrospective study has limitations, as we were not able to identify the stage of MCC among patients. Despite the fact that the controlled patient population was matched exactly by age, race, and gender, we did not account for other confounding variables such as smoking history or income range. Given that the All of Us database is organized according to billing codes, it is possible that cases of certain comorbidities were either not classified or coded incorrectly, resulting in an underestimation of the true odds ratios of these relationships. The All of Us database is 55.2% White, 19.0% Black, 18.1% Hispanic, 3.5% Asian, and 4.2% Other [3], whereas the 2022 US Census racial demographics were 58.9% White, 13.6% Black, 19.1% Hispanic, 6.1% Asian, and 2.3% Other [5], The presence of less representative White and Asian patient populations in this database and a more representative Black patient population may contribute to sampling bias. The database's demographic composition, with a lower representation of White and Asian populations compared to the US Census data, suggests that the findings may not be fully generalizable to the entire US population. This underrepresentation has the potential to overestimate or underestimate the true odds ratios and the intensity of the observed relationships [1,2]. Additionally, although age-matched controls were used, further stratification and analysis within different age groups could potentially provide a more nuanced understanding of the risk gradient for developing BCC, SCC, and melanoma among MCC patients. This approach would help to mitigate the impact of age as a confounding factor, as the incidence of these skin malignancies is known to increase with age.

## Conclusions

This study provides a thorough examination of the relationship between MCC and other skin malignancies, indicating a considerable risk gradient for developing BCC, SCC, and melanoma in MCC patients. The demographic analysis agrees with the known predilection of MCC for elderly Caucasian males, but the study's limitations, including the inability to identify the stage of MCC among patients and the lack of consideration for other confounding variables, call for caution in interpreting the findings.

The possibility of sample bias in the All-of-Us database emphasizes the need to assess the patient population's representativeness in such investigations. Our findings emphasize the importance of additional study to unravel the underlying mechanisms of these correlations and create focused preventative and treatment measures. Future research should seek to overcome the limitations noted in this study, such as taking other potential confounding variables into account and assuring a more representative patient sample.
